# Food Choices and Consequences for the Nutritional Status: Insights into Nutrition Transition in an Hospital Community

**DOI:** 10.1371/journal.pone.0140807

**Published:** 2015-11-11

**Authors:** Jitendra Piple, Ranjeet Gora, Pragati Purbiya, Ashish Puliyel, Parul Chugh, Pinky Bahl, Jacob Puliyel

**Affiliations:** 1 Department of Pediatrics, St Stephens Hospital, Delhi, 110054, India; 2 DocuBuzz Computer Solutions 02-12/25, The Fra*n*klin, 118223, Singapore; 3 Statistician Department of Research, Sir Ganga Ram Hospital, Delhi, India; National Institute of Agronomic Research, FRANCE

## Abstract

**Introduction:**

Although economic development is generally accompanied by improvements in the overall nutritional status of the country’s population the ‘nutritional transition’ often involves a shift to high energy diets and less exercise with negative consequences. This pilot study was done to examine if education of parents operates at the household level to influence dietary choices and the nutritional status of children in a small community of hospital workers.

**Material and Methods:**

3 groups of persons with varying skill and education levels participated. Weighed food logs were used in all households to calculate ‘adult equivalent’ per-capita-consumption. Nutrients were calculated using nutrients calculator software. BMI was used to classify children as underweight, normal weight and overweight.

**Results:**

128 individuals participated from 30 families included 47 children. 10 children (21%) were underweight, 29 (62%) were normal and 8 (17%) were overweight. Energy consumption was highest in families with overweight children 2692 +/-502 compared to 2259 +/-359 in families with normal weight and 2031+/-354 in the family of underweight children. These differences were statistically significant. 42% underweight children belonged to Class 1 at the lowest skill level and there were no overweight children in this group. Most of the overweight children belonged to Class 2. In Class 3 there were no underweight children and the majority was normal weight children.

**Conclusion:**

Underweight children came from the poorer households. Per capita intake of the family as a whole correlated well with BMI in the children. There was increased obesity in middle income families belonging to Class 2—probably in families who move up the scale from deprivation. Nutritional status in children correlated mostly with maternal education status.

## Introduction

Economic growth and human development require well nourished populations who can learn new skills, think critically and contribute to their communities [1]. Economic development is normally accompanied by improvements in a country’s food supply and the gradual elimination of dietary deficiencies, thus improving the overall nutritional status of the country’s population [2]. However this Food and Agriculture Organisation (FAO) report points out that increasing urbanization in poor countries has consequences which are not always positive. In countries with widespread deprivations, when food supply becomes more abundant, there occur changes in diets, patterns of work and leisure—often referred to as “nutrition transition”. The diet shifts towards a higher energy density with a larger role of fat and sugars in foods. The greater saturated fat intake, reduced fruit and vegetable intake and reduced intake of complex carbohydrates and dietary fiber may have negative consequences [3][4]. These dietary changes along with reduced physical activity result in obesity and an epidemic of non-communicable diseases in developing countries [5].

Gaiha and colleagues analyzed the dietary shift and diet quality in India based on the three rounds of National Sample Survey and found that food composition/diet changed considerably in both rural and urban areas over the period 1993–2009. The key features are a reduction in intake of staples (cereal and pulses) and an increase in intake of more energy dense foods, particularly fats [6]. The National Sample Survey office’s (NSSO) 2011–12 data on nutritional intake shows that per capita calorie consumption has risen to 2099 kilocalories per day in rural areas and 2058 kilocalories per day in urban areas[7]. Both numbers are still below India’s Planning Commission benchmark of 2,400 kilocalories per day.

Most studies look at nutrition at the level of the community. In India, on the one hand there are mountains of food grain stocks [8] and on the other hand there are deaths from starvation, and widespread stunting from malnutrition [9]. According to National Family Health Survey 3, almost half of children under age five years (48 percent) are chronically malnourished and stunted [10]. It is evident that persons of very different socioeconomic and educational status live side by side. A study done for the Public Report on Health found that there was great variation in the dietary intake; with energy intakes in some groups double that in others [11]. Looking at nutrition at the community level, in effect, averages the data from the two extremes and this blunts the ability of such studies to investigate issues related to nutritional transition. This is best studied looking at different socioeconomic grouping separately.

This pilot study was done to examine if education of parents operates at the household level to influence dietary choices and nutrition in children in a community of hospital workers. In this study we purposefully selected 3 groups of persons with varying skill and education levels to study their dietary intakes and the effects it had on the nutrition of their children.

## Material and Methods

International Standard Classification of Occupations (ISCO)-88 delineates 4 major groups considering formal education and skill levels. Professionals are put at skill level 4, nurses and associate professionals and technicians’ at skill level 3, clerks and service workers at skill level 2 and elementary occupations needing no special training at skill level 1. To categorize households we used this classification looking at education and skill of the primary breadwinner in the family. Persons belonging to ISCO class 1 (house keeping personnel) class 3 (staff nurses) and class 4 (doctors) at a hospital were selected for the study. For simplification these classes are re-designated Class 1 (house keeping staff) Class 2 (nurses) and Class 3 employees (doctors) for this report. We studied staff working at a hospital each of whom knew the researchers personally and with whom they had a good rapport to ensure compliance with the rigors needed to maintain detailed weighed logs of household food consumption. We studied the per capita dietary intake in the households and the nutritional status of children.

The focus of our study was the nutritional status of children. Body Mass Index (BMI) [weight (in kg)/height(m^2^)] using WHO standards as underweight (BMI < 5^th^ centile) normal (BMI > 5^th^ to <85^th^ centile) or overweight (BMI > 85^th^ centile) were used. Food choices and food intake were studied at the family-unit level to understand what effect dietary choices had on the nutritional status of children.

Kitchen weighting machines (Venus domestic scale, Model KCE, Rajasthan) were provided to each household and all the raw food material used in the kitchen in cooking was recorded on 2 consecutive days. Family details including family members, age, sex, weight, height, education and total family income were also documented. Food logs detailing actual weights of the raw food items used in cooking in the household each day were analysed. The dietary software analysing nutrient content of Indian foods was used to calculate nutrients consumed in the household each day. This dietary software (http://bit.ly/ncalculator) was developed using the nutritive value of Indian foods by Gopalan et al [12]. The software has been validated previously [11].

### Sample size calculation

The previous study using the food log calculator found that per capita energy consumption varied from 1800 kcal to nearly double that value (3500 kcal) between groups. As the variation in energy consumption was so large we assumed that a margin of error of 20% would be acceptable for each group. Looking for differences at 95% confidence interval, we calculated that we would need to study a sample of at least 25 individuals (adult equivalents) in each of the 3 groups [13]. To make allowances for dropouts we targeted a sample size of approximately 40 individuals per group and assuming the average family size of 4 members per family, we planned to study a total 120 individuals in 30 families.

### Statistical analysis

Statistical analysis was performed using the SPSS program for Windows, version 17.0. Continuous variables are presented as mean ± SD, and categorical variables are presented as absolute numbers and percentage. Data were checked for normality before statistical analysis using Shapiro Wilk test. The Kruskal Wallis test was used for the comparison of three or more groups and further comparisons were done using Mann Whitney U test. Categorical variables were analyzed using the chi square test. For all statistical tests, a p value less than 0.05 was taken to indicate a significant difference.

The per capita intake (per adult equivalents) in the household was used for comparison between families. Adult-equivalent conversion factors used previously were employed [14]. The adult-equivalent scale makes allowance for family composition and the presence of family members with distinct energy needs, like children. The food consumed in the household was assumed to be distributed fairly within the family unit, each receiving a share of the household pot in proportion to their age.

The study was approved by the St Stephens Research Committee and the Institutions Ethics Committee of St Stephens Hospital. Written informed consent was taken for participants from the guardian of the children. The consent forms were approved by the Institutional Ethics Committee.

## Results

A total 128 individual (corresponding to 110.42 adult equivalents) participated in the study providing us with food log data. These individuals from 30 families included 47 children. Out of total 47 children, 24 (51.5%) were girls and 23 (49.9%) were boys. The mean age of children is 5.22 ± 2.79 year with median age was 5 year. Our study found 38% children were malnourished, with 21% (10 children) in the sample underweight and 17% (8 children) overweigth/obese and 62% (29 children) were normal using BMI criteria.

Table 1 shows the per capita (adult equivalent) consumption in the household of the underweight, overweight and normal weight children. Energy consumption was highest in families with overweight children (2692 +/-502) compared to 2259 +/-359 in families with normal weight and 2031+/-354 in the family of underweight children. Fat and protein consumption was also correspondingly higher. The households with overweight children were consuming more energy from animal sources (622 calories in the overweight group was from animal sources compared to 342 calories in the underweight group). These differences were statistically significant for total calories, fat and protein consumption.

**Table 1:** The mean per capita intake in households with underweight, normal and overweight children.

		BMI Category			P value
		Underweight (n = 10)	Normal (n = 29)	Overweight/Obese (n = 8)	
Energy (Kcal)	**Mean ± SD [Median]**	2031.29 ± 354.21 [1978.63]	2259.21 ± 359.15 [2298.63]	2692.4 ± 501.86 [2745.28]	0.019
Carbohydrates(g)	**Mean ± SD [Median]**	304.35 ± 73.56 [274.11]	389.54 ± 398.76 [304.79]	598.58 ± 748.23 [334.8935]	0.202
Protein (g)	**Mean ± SD [Median]**	58.81 ± 12.64 [54.11]	66.3 ± 11.9 [65.52]	88.19 ± 22.73 [80.38]	0.001
Fat (g)	**Mean ± SD [Median]**	58.57 ± 17.17 [55.94]	74.81 ± 22.62 [79.03]	108.51 ± 28.49 [113.45]	0.001
Energy(kcal) animal source	**Mean ± SD [Median]**	323.6 ± 157.34 [305.38]	497.48 ± 209.8 [534.01]	622.42 ± 191.75 [551.46]	0.022
Crude Fiber (g)	**Mean ± SD [Median]**	6.97 ± 2.4 [6.48]	8.66 ± 4.86 [7.44]	7.74 ± 1.95 [7.625]	0.471
Minerals (g)	**Mean ± SD [Median]**	11.65 ± 2.89 [11.00]	13.28 ± 2.38 [12.86]	15.26 ± 2.64 [14.74]	0.014
Iron (mg)	**Mean ± SD [Median]**	15.68 ± 4.34 [14.72]	18.91 ± 10.5 [16.25]	18.37 ± 5.52 [16.72]	0.621
Calcium (mg)	**Mean ± SD [Median]**	756.08 ± 262.04 [671.55]	1072.59 ± 362.16 [1137.4]	1198.17 ± 386.66 [1146.185]	0.038
Phosphorus (mg)	**Mean ± SD [Median]**	1517.25 ± 336.99 [1425.39]	1684.78 ± 247.7 [1670.32]	2035.92 ± 358.72 [2015.63]	0.005
Carotene (mcg)	**Mean ± SD [Median]**	1251.35 ± 1108.18 [491.95]	1787.17 ± 1886.56 [642.25]	695.67 ± 288.51 [599.505]	0.311
Thiamine (mcg)	**Mean ± SD [Median]**	1.45 ± 0.4 [1.27]	1.49 ± 0.36 [1.466]	1.65 ± 0.4 1.7]	0.381
Riboflavin (mg)	**Mean ± SD [Median]**	0.64 ± 0.24 [0.61]	1.01 ± 0.34 [1.08]	1.04 ± 0.9 [1.045]	0.004
Niacin (mg)	**Mean ± SD [Median]**	14.6 ± 4.33 [13.09]	14.13 ± 2.64 [13.88]	15.44 ± 3.56 [15.12]	0.800
Total B6 (mg)	**Mean ± SD [Median]**	0.16 ± 0.09 [0.13]	0.16 ± 0.14 [0.14]	0.12 ± 0.1 [0.114]	0.401
Folic acid (free) (mcg)	**Mean ± SD [Median]**	64.48 ± 16.29 [63.24]	71 ± 15.33 [67.99]	89.4 ± 22.53 [87.065]	0.041
Folic acid (total) (mcg)	**Mean ± SD [Median]**	193.71 ± 53.75 [176.92]	210.88 ± 67.58 [202.20]	283.82 ± 93.51 [267.415]	0.031
Vitamin C (mg)	**Mean ± SD [Median]**	74.05 ± 49.51 [53.41]	50.38 ± 29.91 [50.87]	47.68 ± 23.55 [37.28]	0.443

The impact of socio-economic position (SEP) on children’s weight-status was assessed using four indicators of SEP: skill level classification (Table 2), monthly per capita income (Table 3), mother’s education (Table 4) and father’s education (Table 5).

**Table 2:** Nutritional status of children in different skill level classes.

Skill level Classification	BMI Category		
	Underweight Frequency (%)	Normal Frequency (%)	Overweight/Obese Frequency (%)
Class 1	8 (42%)	11 (58%)	0 (0%)
Class 2	2 (14%)	7 (50%)	5 (36%)
Class 3	0 (0%)	11 (79%)	3 (21%)
Total	10 (21%)	29 (62%)	8 (17%)

P value 0.006

**Table 3:** Nutritional status against per capita income of household.

Monthly per capita income	BMI Category		
	Underweight Frequency (%)	Normal Frequency (%)	Overweight/Obese Frequency (%)
Rs 5000 or less	8 (42%)	11 (58%)	0 (0%)
Rs 5000–19999	2 (10%)	14 (70%)	4 (20%)
Rs 20000 and over	0 (0%)	4 (50%)	4 (50%)
Total	10 (21%)	29 (62%)	8 (17%)

P value 0.004

**Table 4:** Nutritional status of children against maternal education levels.

Mother Education	BMI Category		
	Underweight Frequency (%)	Normal Frequency (%)	Overweight/Obese Frequency (%)
Secondary school education or less(Support staff)	8 (40%)	12 (60%)	0 (0%)
Graduate Mothers(Nurses)	2 (13%)	6 (40%)	7 (47%)
Post Graduate Mothers(Doctor families)	0 (0%)	11 (92%)	1 (8%)
Total	10 (21%)	29 (61%)	8 (17%)

P value 0.005

**Table 5:** Nutritional status of children against paternal education levels.

Father’s Education	BMI Category		
	Underweight Frequency (%)	Normal Frequency (%)	Overweight/Obese Frequency (%)
Secondary school education or less	6 (30%)	12 (60%)	2 (10%)
Graduate	4 (29%)	7 (50%)	3 (21%)
Post Graduate	0 (0%)	10 (77%)	3 (23%)
Total	10 (21%)	29 (62%)	8 (17%)

P value 0.068

When the children were categorized according to ISCO classification there were no overweight children in the Class 1 group and most of the overweight children were from the class 2 group. (Table 2)

Table 3 looks at the income of the households. There were no overweight children in the families with per capita income (PCI) less than Rs 5000/month but 8 were underweight. There were no underweight children families with PCI over Rs 20,000/months but 4 were overweight. The differences between groups were statistically significant.

Table 4 shows effect of mothers’ education level on BMI. 20 children were born to mother who have studied up to secondary school or less. 8 of them were underweight and there were none who were overweight. 15 children were born to graduate mothers and 7 of them were overweight. Among 12 children of mothers who had completed post graduate education there was no child who was underweight and only 1 was overweight.

Table 5 shows nutrition of children against education levels of fathers. It shows a similar trend, but the differences are less stark and do not achieve statistical significance.

The raw data on food consumption and nutrient intake is available in S1 Table.

## Discussion

Popkin has lamented that there are “major gaps in this literature related to lack of data and superficial examinations of patterns and trends without sufficient attention to the extant literature and the dynamics of change rather than simplistic cross-sectional perspectives” [4]. We purposefully selected to study children in 3 groups, whose parent’s education and skill levels and the per capita incomes were different. The purpose of our study was to study diets in households at different categories of education and skill levels to address one aspect of this gap.

Our study found 38% children were malnourished, with 21% in the sample underweight and 17% overweigth/obese and this exemplifies the ‘double burden of malnutrition’ in India described by Ravishankar [15].

We found that BMI of children was related to the per capita calorie consumption in the household. Households with overweight children were consuming more energy, proteins and fats and were getting more calories from animal sources. Urban children are given to food fads and a tendency to consume ‘junk food’—rich in calories from sugar and fat with little protein, fiber, vitamins or minerals and this is often blamed for the increasing obesity seen among the youth. Our study suggests that obesity is related to the household diet and in a way shifts the blame from children to the parents.

Underweight was most common in children of the lowest paid (Class 1). 42% of children in this group were underweight and they constituted 80% of all the underweight children. There was no child who was overweight in the group. The majority of overweight children (62.5%) were children in Class 2. Although there was more affluence in the Class 3 category, they accounted for only 37.5% of obesity in children.

The large number of underweight children among Class 1 families suggests that economic constraints limit calorie intake in this group. The Government of India has defined the ‘poverty line’ as the bare minimum income needed to provide basic food requirements, based on the costs of food. It does not account for other essentials such as health care and education [16]. The Rangarajan panel on poverty in India has suggested to the government that those spending more than Rs 972 a month in rural areas and Rs 1,407 a month in urban areas in 2011–12 are above this poverty threshold [17]. In groups who live on a sub subsistence income per capita income (PCI) and per capita expenditure can be used interchangeably as they have nothing to save. The lowest PCI in the families we studied was Rs 2000 and this is well over the poverty threshold described above. The poverty line may need to be raised if this threshold is meant to indicate economic disadvantage, to identify individuals and households in need of government assistance and aid, to prevent chronic malnutrition and stunting.

Intuitively we would expect wealthier Class 3 families (education and skill levels 4 with the highest per capita income) to be eating richer foods which are more energy dense. However we found more overweight children among Class 2 households (education and skill level 3) and they consume more calories, protein and fat. Although not formally documented in this study, it is known that many of the nurses who are now in Class 2 had, in their lifetimes moved up from class 1 through education and skill development. We can speculate that these mothers originally from poor backgrounds may tend to overcompensate, so that their children do not suffer the deprivations they suffered in their childhood.

This finding is reminiscent of the finding of Griffiths et al in the UK who looked at the middle class separately from upper and lower class children and found that middle-class children are more likely to be obese than those from the other groups [18]. They found that for all measures of adiposity the highest probability of being classified as obese is in the middle of the English Indices of Deprivation [19]. Griffiths reported that parents of lower middle families are busy with their work and their basic priorities are children educational achievement and material possession. They enjoy food, and believe themselves to have knowledge about healthy eating. Although the whole household is likely to be overweight Griffiths suggest that parents do not recognize the problem.

It is known that obesity increases in developing economies, but is less in stable economies (regardless of whether stability is achieved at low development levels or in more developed economies) [20, 21]. This trend seems also to be reflected at the household level. Families of mothers who had moved up in social class in their own lifetime are particularly vulnerable to have problems with obesity in their children.

We found that normal nutrition was best correlated with mother’s education and the better educated mothers have the better chance of rearing normal weight children. Similar to our findings Abuya et al in Nairobi slums found stunting in 40% of children and that mother’s education was a strong predictor of child’s nutritional status in urban slum settings [22].

Our study is based on a population of workers in a hospital setting. This study focusing on one group of highly motivated staff helped improve the internal validity of the findings but the generalisability to the wider community must yet be tested. Also hospital staff is known to work in shifts and children are often cared for by persons who are not the parents of the child. This can affect reproducibility of these findings in other settings. We hope that our findings will help hypothesis generation looking at the underlying dynamics of nutritional transition.

## Conclusion

Using cross sectional data, nutritional transition is said to be responsible for the increase in obesity seen in lower and middle income countries with increasing urbanization. At the level of the household, our data shows that the same nutritional transition results in increasing obesity in middle income families probably as families move up the scale from deprivation.

## Supporting Information

S1 Table The raw data on food consumption and nutrient intake. (XLSX)
